# Eggshell and Feed Microbiota Do Not Represent Major Sources of Gut Anaerobes for Chickens in Commercial Production

**DOI:** 10.3390/microorganisms9071480

**Published:** 2021-07-11

**Authors:** Jiri Volf, Magdalena Crhanova, Daniela Karasova, Marcela Faldynova, Tereza Kubasova, Zuzana Seidlerova, Alena Sebkova, Michal Zeman, Helena Juricova, Jitka Matiasovicova, Marian Foltyn, Zdenek Tvrdon, Ivan Rychlik

**Affiliations:** 1 Veterinary Research Institute, Hudcova 70, 621 00 Brno, Czech Republic; volf@vri.cz (J.V.); crhanova@vri.cz (M.C.); karasova@vri.cz (D.K.); faldynova@vri.cz (M.F.); kubasova@vri.cz (T.K.); seidlerova@vri.cz (Z.S.); asebkova@vri.cz (A.S.); zeman@vri.cz (M.Z.); juricova@vri.cz (H.J.); matiasovicova@vri.cz (J.M.); 2 Hatchery Vodnanske Kure, Komenskeho 75, 768 11 Chropyne, Czech Republic; marian.foltyn@vodnanskekure.cz (M.F.); zdenek.tvrdon@vodnanskekure.cz (Z.T.)

**Keywords:** chicken, microbiota, caecum, eggshell, hatchery, feed

## Abstract

In this study, we addressed the origin of chicken gut microbiota in commercial production by a comparison of eggshell and feed microbiota with caecal microbiota of 7-day-old chickens, using microbiota analysis by 16S rRNA sequencing. In addition, we tested at which timepoint during prenatal or neonatal development it is possible to successfully administer probiotics. We found that eggshell microbiota was a combination of environmental and adult hen gut microbiota but was completely different from caecal microbiota of 7-day-old chicks. Similarly, we observed that the composition of feed microbiota was different from caecal microbiota. Neither eggshell nor feed acted as an important source of gut microbiota for the chickens in commercial production. Following the experimental administration of potential probiotics, we found that chickens can be colonised only when already hatched and active. Spraying of eggs with gut anaerobes during egg incubation or hatching itself did not result in effective chicken colonisation. Such conclusions should be considered when selecting and administering probiotics to chickens in hatcheries. Eggshells, feed or drinking water do not act as major sources of gut microbiota. Newly hatched chickens must be colonised from additional sources, such as air dust with spores of Clostridiales. The natural colonisation starts only when chickens are already hatched, as spraying of eggs or even chickens at the very beginning of the hatching process did not result in efficient colonisation.

## 1. Introduction

Birds or mammals are hatched or born nearly sterile, but immediately after birth they are exposed to an external environment, rich in different forms of life. Interactions between ubiquitously present prokaryotes and new-borns are frequent. Age-dependent colonisation of different animal body compartments by prokaryotic microbiota has been repeatedly described, including chickens and the chicken intestinal tract [1,2,3,4]. The intestinal tract of chickens is first colonised by *E. coli*, which is characteristic for chicken gut microbiota during the first week of life. *E. coli* is replaced by bacteria from phylum Firmicutes during the second week of life, mostly by aerotolerant Lactobacillaceae and spore-forming isolates from the Lachnospiraceae, Ruminococcaceae and Clostridiaceae families. Representatives of phylum Bacteroidetes and strict anaerobes from phylum Proteobacteria are usually among the last ones to appear in gut microbiota of chickens in commercial production. A commonly overlooked fact is that, due to the absence of hens in egg incubation and hatching, the development of gut microbiota in chickens in commercial production is highly specific, resulting in microbiota found in the caecum of one-week-old chickens originating exclusively from the environment [4,5]. If adult hens were present, the developmental pattern would be different [6]. However, if hens are excluded from this process, what is the origin bacterial species colonising chicken caecum in the first weeks of life?

This topic was addressed in the current study. Specifically, we asked two questions. Firstly, considering that the transmission of microbiota from hens to offspring is interrupted in commercial production, where do members of Lachnospiraceae or Ruminococcaceae families come from, and why do they appear in the chicken intestinal tract so shortly after hatching? Do they originate from eggshells, feed or drinking water? Secondly, at what timepoint during prenatal or neonatal development is it possible to colonise the chicken intestinal tract? Answers to these questions are of utmost importance because the rapid establishment of complex gut microbiota is crucial for chicken resistance to enteric infections [7,8,9]. To address these questions, we compared eggshell microbiota with gut microbiota of 7-day-old chicks. Next, we tested whether similar or different gut microbiota would develop in chickens hatched from eggs originating from different parent flocks, i.e., with different microbiota deposited on eggshells. We also investigated to what extent feed and drinking water act as a source of gut microbiota. Finally, we tested at what timepoint during egg incubation or hatching it is possible to administer bacterial strains to achieve successful gut colonisation.

## 2. Materials and Methods

### 2.1. Comparison of Eggshell Microbiota with Caecal Microbiota of 7-Day-Old Chicks

Forty-five fertilised eggs of ISA Brown egg laying line were obtained from a local hatchery. These eggs originated from 3 different reproductive flocks from different geographical locations in the Czech Republic (15 eggs per flock). Three eggs from each batch were used for DNA purification from eggshells and the remaining 12 eggs from each flock were incubated in a small-scale hatching incubator. Since the hatching efficiency in the small-scale experiment was around 50%, five, five and four chickens were obtained from each parent flock, respectively. The chickens were labelled by leg rings and raised in the same cage. The chickens were sacrificed at the age of one week and caecal contents were collected for microbiota characterisation by sequencing the V3–V4 variable region of 16S rRNA genes.

### 2.2. Housing Conditions

Chickens were kept in fully air-conditioned barrier rooms, in plastic boxes with slatted floors. Feed and drinking water were supplied ad libitum. Temperature was set to 34 °C on day 1; this decreased by 2 °C per day. In addition, the boxes were equipped with brooders. A light regime started with 24 h light on day 1, followed by 22 h light and 2 h dark on day 2, 20 + 4 h on day 3, 19 + 5 h on day 4, and 18 + 6 h on day 5, and so on.

### 2.3. DNA Purification from Eggshells

Three visually clean eggs from each farm were used for DNA purification from eggshells according to [10], as follows: The inner and outer shell membranes were removed, and 50 mg of eggshell was placed in a 2 mL tube with 2.5 mm zirconium silicate beads and 1600 µL of 0.5 M EDTA. The shells were disrupted in a MagnaLyser (Roche, Basel, Switzerland) at 7000 rpm for 60 s, followed by overnight incubation on a shaker heated to 44 °C. Finally, DNA was purified from the supernatant using the DNeasy tissue kit and following the instructions of the manufacturer (Qiagen, Hilden Germany).

### 2.4. Feed as a Source of Gut Microbiota

Two groups of ISA Brown chickens with 4 birds per group were formed (different from the chickens used above for comparison of caecal and eggshell microbiota). The control group was provided with conventional feed and water throughout the whole experiment. The experimental group was provided with a fresh batch of autoclaved (121 °C for 20 min) feed and water on a daily basis. The two groups of chickens were kept in the same room, but in two different cages separated by 2 m distance to minimise any group separation effect. The experiment was terminated when the chickens were one week old. The body weight of all chickens was determined and caecal contents were collected during post-mortem analysis. In addition, a single sample of feed before (conventional feed) and after (sterile feed) autoclaving was saved for DNA purification and feed microbiota characterisation. To test the viability of bacteria in the conventional feed, an aliquot of the feed was homogenised, serially diluted in PBS, and each dilution was plated on WCHA agar plates. The agar plates were incubated anaerobically at 37 °C for 48 h and the bacterial mass was washed with 5 mL of PBS from the dilution plate with approx. 1000 growing colonies. Washed bacteria were pelleted by centrifugation at 14,000× *g* for 1 min and the pellet was used for DNA purification.

### 2.5. Definition of Optimal Age for Administration of Bacterial Strains Intended for Gut Colonisation

Forty-eight fertilised eggs of ISA Brown line were obtained and assigned to 3 groups with 16 eggs per group. The first group of eggs remained untreated throughout the whole experiment. The second set of 16 eggs was sprayed on day 1 of egg incubation with a mixture of *Pseudoflavonifractor capillosus*, *Anaerotruncus colihominis*, *Butyricicoccus pullicaecorum*, *Blautia producta*, *Clostridium lactatifermentans*, *Clostridium glycyrrhizinilyticum* and *Oscillibacter valericigenes*. These bacterial species were selected as spore-forming species that might be able to survive as spores on the eggshells [11]. The last group of 16 eggs was treated with the same bacterial mixture on day 1 and the treatment was repeated on day 20 of embryonic development. Eight chickens from control group eggs, nine chickens treated once in the beginning of embryonic development and 12 chickens treated twice during embryonic development were obtained and sacrificed one week after hatching. Caecal contents were collected, DNA from caecal contents was purified and bacteria present in the mixture were quantified by strain-specific real-time PCR (see below).

In the second experiment of this type, *Megamonas hypermegale* and *Bacteroides caecicola* were used for spraying eggs and hatching chickens of Ross 308 line. These bacterial strains were selected as capable of efficient colonisation of the chicken caecum [12]. A mixture of these isolates was sprayed over the tray with 50 eggs on day 21 of embryonic development, either at the moment when the first chickens just began to hatch (when less than 10% of chickens were already hatched), or 10 h later, when 90% of chickens were already hatched. Five hatched chickens were taken from each group together with 5 control, non-treated chicks. These chickens were sacrificed when one week old, caecal contents were collected, DNA was purified and the presence of bacteria from the mixture was determined by strain-specific real-time PCR. This experiment was repeated twice.

### 2.6. DNA Purification

All collected samples were stored at −20 °C before DNA purification and the storage never exceeded 3 months, to minimise sample degradation. Each sample was homogenised using a MagnaLyser (Roche, Basel, Switzerland) and the homogenate was subjected to DNA purification. QIAmp DNA Stool kit (Qiagen, Hilden, Germany) was used for DNA purification from caecal contents, while DNeasy tissue kit (Qiagen, Hilden, Germany) was used for DNA purification from eggshell or bacterial mass collected from agar plates. Purified DNA was stored at −20 °C.

### 2.7. Quantitative Real-Time PCR

Strain-specific primers (Table 1) were designed as described previously [13]. Real-time PCR was performed in 3 µL volumes in 384-well microplates using QuantiTect SYBR Green PCR Master Mix (Qiagen, Hilden, Germany) and a Nanodrop pipetting station (Innovadyne, Santa Rosa, CA, USA) for dispensing PCR mixtures, as reported elsewhere [13].

### 2.8. Microbiota Characterisation by Sequencing of V3/V4 Variable Region of 16S rRNA Genes

The concentration of purified DNA was determined spectrophotometrically and DNA samples diluted to 5 ng/mL were used as a template in PCR with forward primer 5′-TCGTCGGCAGCGTCAGATGTGTATAAGAGACAG-MID-GT-CCTACGGGNGGCWGCAG-3′ and reverse primer 5′-GTCTCGTGGGCTCGGAGATGTGTATAAGAGACAG-MID-GT-GACTACHVGGGTATCTAATCC-3′. MIDs represent different sequences of 5, 6, 7, or 9 nucleotides in length, which were used to identify individual samples in the sequencing run. PCR amplification was performed using a KAPA HiFi Hot Start Ready Mix kit (Kapa Biosystems, Woburn, MA, USA) and the resulting PCR products were purified using AMPure beads. Sequencing was performed using MiSeq Reagent Kit v3 (600 cycle) and MiSeq apparatus according to the manufacturer’s instructions (Illumina, San Diego, CA, USA), as described previously [6]. The fastq files were imported into QIIME software [14]. Forward and reverse sequences were joined, and chimeric sequences were predicted by the slayer algorithm and excluded from downstream analyses. The non-chimeric sequences were classified by RDP Seqmatch with an operational taxonomic units (OTU) discrimination level set to 97%. Principal coordinate analysis (PCoA) implemented in QIIME was used for data visualisation.

### 2.9. Statistics and Data Availability

The Mann–Whitney test was used to identify differently abundant taxa among the samples. A *t*-test was used for comparison of body weights of chickens fed either a conventional or a sterile diet. Differences with *p* < 0.05 were considered as significant.

## 3. Results

### 3.1. Comparison of Eggshell Microbiota and Caecal Microbiota of 7-Day-Old Chicks

**Figure 1:**
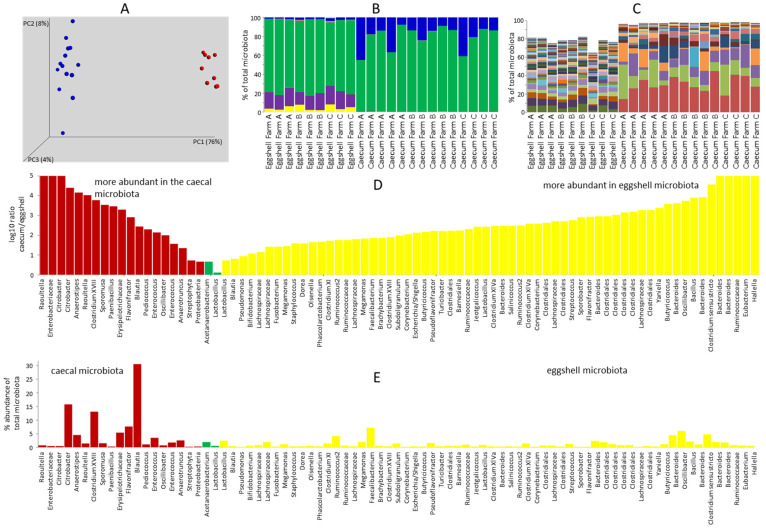
Comparison of eggshell microbiota and caecal microbiota of one-week old chicks. (**A**), weighted Principal Coordinate Analysis (PCoA) of caecal microbiota of one-week-old chickens (blue dots) and microbiota of eggshells (red dots). (**B**), microbiota composition of individual caecal and eggshell samples at phylum level (blue—Proteobacteria, green—Firmicutes, magenta—Bacteroidetes, yellow—Actinobacteria). Microbiota of eggshells resembled caecal microbiota of adult hens due to the presence of Bacteroidetes and Actinobacteria and low representation of Proteobacteria. (**C**), individual samples at OTU level (top 100 OTUs were used for figure generation) showing completely different composition of caecal microbiota of one-week-old chickens and eggshells microbiota before incubation (see Table S1 for OTU composition). (**D**), absolute values of log_10_ ratios of abundances of OTUs forming more than 0.3% of total microbiota, either in the caecum or in eggshells. Since the biological meaning of the ratios in (**D**) is influenced also by abundance in of each microbiota member, either in the caecal or eggshell microbiota, these are shown in (**E**). Abundance in caecal microbiota is shown for the OTUs more abundant in the caecum, and vice versa, abundance in eggshell microbiota is shown for the OTUs more abundant in eggshell microbiota.

Caecal microbiota of 7-day-old chickens differed from eggshell microbiota (Figure 1A). Microbiota of 7-day-old chickens consisted of Firmicutes and Proteobacteria. On the other hand, eggshell microbiota consisted of representatives of Firmicutes, Bacteroidetes, Actinobacteria and a low representation of Proteobacteria (Figure 1B). The differences were also demonstrated at the OTU level (Figure 1C). When the abundance of OTUs, which formed at least 0.25% of either caecal or eggshell microbiota, was compared, only two OTUs were present in similar abundance in the microbiota of 7-day-old chickens and eggshells, while 72 OTUs were specific to either type of sample (Figure 1D,E).

The caecal microbiome of 7-day-old chickens was characteristic for this particular age [1,4,5]. Eggshell microbiota was a mixture of adult hen microbiota and environmental microbiota (Table S1). Eggshell microbiota was dominated by microbiota deposited by the hen during egg laying, since *Faecalibacterium prausnitzii* and *Oscillibacter ruminantium* formed 7.20% and 5.98% of total eggshell microbiota, respectively. In addition, different *Bacteroides* species formed 12% of total microbiota, and *Megamonas hypermegale* and *M. funiformis* formed 1.20% and 1.94% of all eggshell microbiota, respectively. These are all microbiota members characteristic for adult hens. The third most abundant OTU of eggshell microbiota was represented by *Clostridium sporogenes* forming 4.79% of eggshell microbiota. In addition, Actinomycetales (genera *Corynebacterium*, *Brachybacterium* or *Yaniella*) formed 1.40% of eggshell microbiota, indicating the environment as a source [15,16]. *Bacillus coagulans* formed 2.13% of total eggshell microbiota and Staphylococcaceae (genera *Jeotgalicoccus*, *Salinicoccus* and *Staphylococcus*) formed 1.09% of eggshell microbiota, further showing the contribution of the environment [17,18,19,20].

### 3.2. Gut Microbiota of 7-Day-Old Chickens Originating from Different Parent Flocks

**Figure 2:**
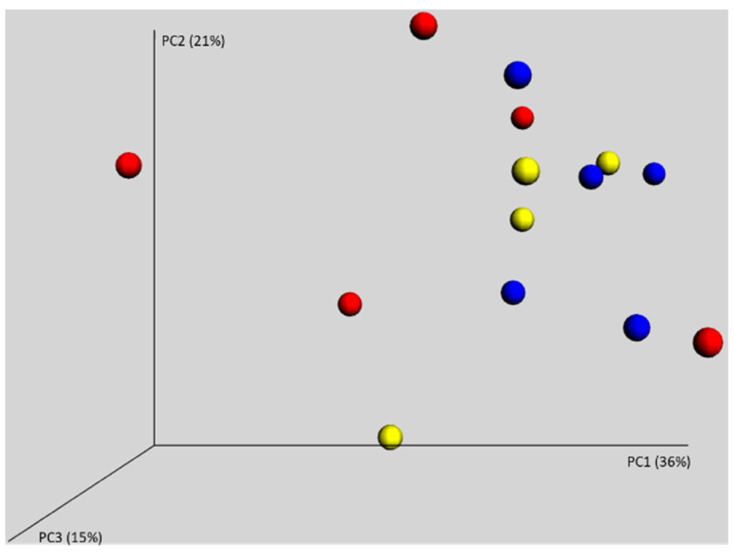
Weighted PCoA of caecal samples of chickens from three different parent flocks. No clustering according to origin of chickens defined by different colours was observed. Parents, therefore, did not play any role in the establishment of gut microbiota in the chickens in commercial production.

Next, we analysed the microbiota composition in the caeca of chickens originating from three different parent flocks, which were hatched and raised together during the first week of life. PCoA showed that all the chickens were randomly distributed and there was no clustering according to origin of the reproductive parent flock (Figure 2). The chickens were, therefore, colonised from environmental sources and not by the microbiota of their (different) parents.

### 3.3. Feed as a Source of Gut Microbiota

**Figure 3:**
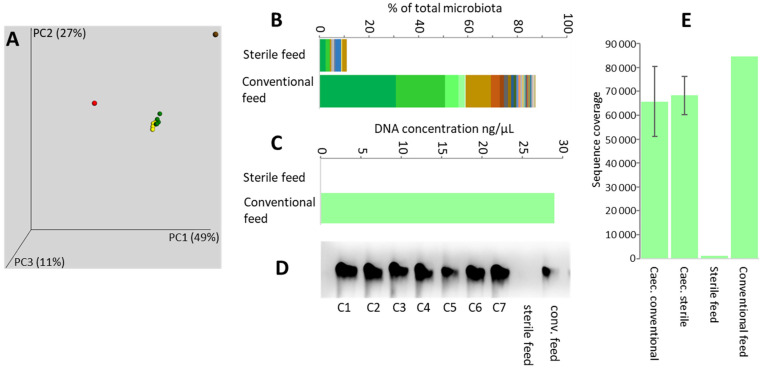
Feed as a source of chicken gut microbiota. (**A**), weighted PCoA analysis of microbiota in conventional (brown dot) and sterile feed (red dot) differed from the caecal microbiota of one-week-old chickens fed with conventional (green dots) or sterile feed (yellow dots). Conventional feed microbiome was dominated by chloroplast rRNA sequences from plants used for feed manufacturing. Top 30 OTUs from conventional feed showed in (**B**) formed 87.3% of all reads, and chloroplast assigned reads (shown in shades of green) formed 59.1% of all reads (see Table S2 for taxa composition). DNA in feed samples was degraded by autoclaving and low DNA concentration (**C**), low quality of PCR products ((**D**), c1–c7 stands for 7 caecal samples, followed by amplification products obtained using DNA from sterile or conventional feed as template) and low number of sequences (**E**) were obtained for the sterile feed sample.

Next, we tested the role of feed in the development of caecal microbiota during the first week of life. Two groups of chickens were provided with either autoclaved feed (sterile feed) or the same feed without autoclaving (conventional feed). Caecal microbiota of both groups of chickens was similar and differed from feed microbiota (Figure 3A). Characterisation of feed microbiota was complicated by the fact that sequences corresponding to plant chloroplast rRNA formed 59.1% of all sequences obtained for the conventional feed sample (Figure 3B). In sterile feed, the DNA including the chloroplast DNA was degraded; it was impossible to purify any DNA from this type of sample (Figure 3C) and obtain 16S rRNA amplification products (Figure 3D). Consequently, the number of reads obtained for the sterile feed sample was extremely low (Figure 3E) and OTU composition in sterile feed must be considered with great care.

**Figure 4:**
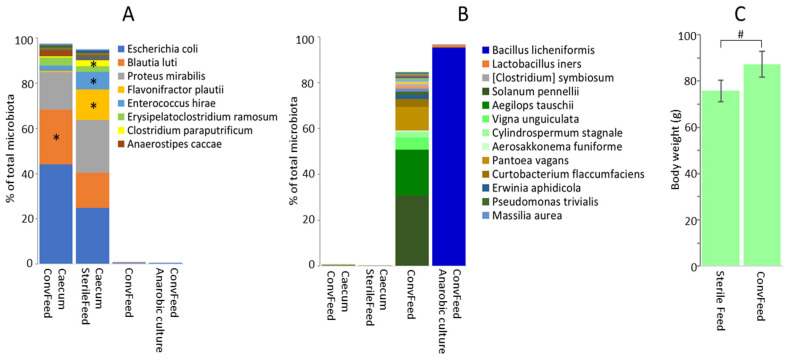
Influence of conventional and sterile feed on caecal microbiota composition and body weight of chickens. (**A**), Composition of caecal microbiota in the chickens was affected by conventional or sterile feed. Asterisks indicate OTUs differently abundant in microbiota of chickens fed conventional or sterile feed. (**B**), Feed microbiota consisted of chloroplast 16S rRNA (different shades of green in conventional feed) and the only viable bacterial species in the feed was *Bacillus licheniformis*. Feed, therefore, was not the main source of gut microbiota for chickens. Autoclaving resulted in degradation of plant chloroplast DNA and negatively affected nutritional quality, which influenced microbiota composition (**A**) but also chicken body weight (**C**). *—significantly different in microbiota of chickens provided with conventional or sterile feed by Mann–Whitney test. #—significantly different by *t*-test.

Microbiota of chickens fed a conventional or sterile diet, despite qualitative similarities, exhibited quantitative differences at the OTU level. Whether chickens were fed with conventional or sterile feed, the top 15 OTUs represent more than 90% of total microbiota for both groups. Out of these, *Anaerostipes caccae* was significantly more abundant in the microbiota of chickens fed conventional feed, while *Flavonifractor plautii*, *Enterococcus hirae* and *Clostridium paraputrificum* were more abundant in the microbiota of chickens fed sterile feed (Figure 4A). The same OTUs represented minor fractions in conventional feed microbiota and in the washes from anaerobically incubated WCHA agar plates inoculated with serially diluted homogenates of conventional feed. Similarly, OTUs common in the conventional feed (*Pantoea vagans*, *Curtobacterium flaccumfaciens*, *Erwinia aphidicola*, *Pseudomonas trivialis*, *Stenotrophomonas rhizophila*, *Sphingomonas olei*) or in bacterial pools washed from agar plates after anaerobic culture (*Bacillus licheniformis*) did not efficiently colonise the chicken caecum (Figure 4B). All results suggest that feed did not act as the major source of gut microbiota, although autoclaving of feed affected its nutritional values and allowed for a different microbiota composition to develop in the chickens. The effect of autoclaving feed was confirmed by the lower body weight of chickens provided with the autoclaved feed in comparison to those fed conventional feed (Figure 4C).

### 3.4. Definition of an Optimal Time Point for Administration of Bacterial Strains during Hatching

**Figure 5:**
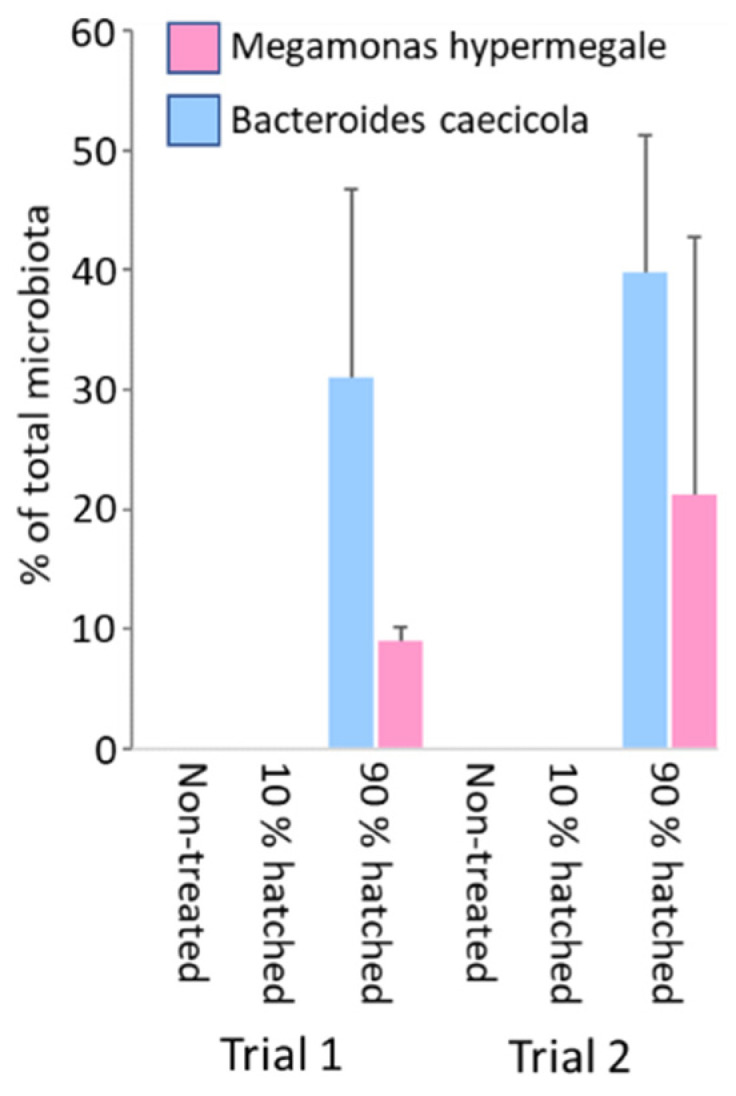
Definition of the timepoint when chickens can be colonised by experimentally administered bacterial strains. Hatching eggs were sprayed with a mixture of *Bacteroides caecicola* and *Megamonas hypermegale,* either when 10% of chickens were already hatched or when 90% of chickens were hatched. Five control chickens were not treated at all. All tested chickens became efficiently colonised only when the majority of them were already hatched and became active.

Finally, we attempted to fine the timepoint during prenatal or neonatal development at which it is possible to efficiently colonise chickens. A mixture of seven different spore-forming species was sprayed over the eggs on day 1 of embryonic development, or both on day 1 and day 20 of embryonic development. When the caecal microbiota composition was determined in one-week-old chicks, none of the strains used for egg treatment were detected in the chicken caecum (Table S3). Next, we used an additional two strains, *Megamonas hypermegale* and *Bacteroides caecicola*, which efficiently colonised the chicken caecum [12]. A mixture of these two isolates was sprayed over the box with incubated eggs on day 21 of embryonic development, either at the moment when less than 10% of chickens were already hatched, or approximately 10 h later, when 90% of chickens were hatched. No colonisation was recorded when the boxes with eggs and a few already hatched chickens were treated. In contrast, successful colonisation of all tested chickens was observed when the tray was sprayed when 90% of chickens were already hatched (Figure 5).

## 4. Discussion

In this study, we addressed the origin of microbiota that appears in the chicken caecum shortly after hatching. Although hens deposit their microbiota on eggshells and adult-type microbiota was found on the eggshells [1,21,22,23], this was not vertically transmitted. The fact that parents do not act as a source of gut microbiota for newly hatched chickens in commercial production was also confirmed by hatching chickens from eggs originating from different parent flocks. Samples from these chickens did not form separate clusters, their microbiota was similar, and these chickens, therefore, had to be colonised from the same environmental sources after hatching. The fact that transmission via eggshell deposition is not efficient was confirmed also by experimental deposition of spore-forming Clostridiales onto the eggshell, either at the beginning of egg incubation or a day before hatching.

The eggshells were contaminated by Actinomycetales and Staphylococcaceae. These bacteria are common in chicken litter [15,16,24] or in the air of poultry houses [18,25] and are known from previous reports to contribute to eggshell microbiota [26,27,28]. Whether viable or not, these were definitively not transferred to the chicken intestinal tract. Feed and drinking water also did not act as a major source of gut microbiota. There were variations in quantities of different OTUs in chickens fed conventional and sterile feed, but qualitatively the same major OTUs were present in microbiota of both groups of chicks. When checking for viable anaerobic bacteria in conventional feed, *Bacillus licheniformis* dominated, followed by *Lactobacillus iners* and *Clostridium symbiosum*, but none of these bacteria colonised the chicken intestinal tract. The second most common OTU in the caeca of one-week-old chicks, *Blautia luti*, formed 0.16% of total feed microbiota, which does not exclude its feed origin. However, *Bl. luti* did not grow after anaerobic culture of feed samples, which suggests that it was present in the feed in inactivated form. Moreover, the eight most abundant anaerobes from the caeca of chicks, *Flavonifractor plautii*, *Erysipelatoclostridium ramosum*, *Clostridium paraputrificum*, *Anaerostipes caccae*, *Clostridium perfringens*, *Clostridium saccharogumia*, *Pediococcus acidilactici* and *Romboutsia timonensis,* each formed 0.02% or less of the total feed microbiota, and except for *Romboutsia timonensis*, none of these OTUs were detected in washes from anaerobic cultures. Feed, therefore, does not represent a major source of gut microbiota for chickens in commercial production although feed shapes gut microbiota composition, as observed in the chickens fed with conventional and sterilised feed.

Finally, we tested at what time during the hatching period it is possible to administer gut microbiota and achieve successful colonisation. Spraying of eggs or even chickens at the very beginning of the hatching process, when the majority of chickens were still in eggs, did not result in efficient colonisation. This also means that spraying of eggs any time during incubation with strict anaerobes, i.e., before the hatching, will not result in efficient colonisation. Chickens could be colonised only when already hatched, not considering in ovo inoculation with facultative anaerobes [29,30]. The early administration of probiotics, therefore, results in treatment failure, while delayed treatment of chickens with complex microbiota is also not recommended since such products do not act therapeutically [7]. Therefore, the window for reasonable administration is quite short, lasting for a few hours at hatching, before placement on a farm where chickens may become colonised by microbiota of unintended composition. Although this window is rather short, it is still long enough to logistically arrange the whole production process and include probiotic treatment at an appropriate time and place.

## Figures and Tables

**Table 1:** List of primers used for the detection and quantification of target bacterial species.

Target Species	Forward Primer	Reverse Primer
*Pseudoflavonifractor capillosus*	GCCAGGAATTTCCCTTCTTC	GGCAGTCTCATGGAGGTAGC
*Anaerotruncus colihominis*	GCAAATGGGCGTATCCTCAA	CTGTTCAATTTCCCGGCAC
*Butyricicoccus pullicaecorum*	CGAGCAGGCAAACGACAA	CCAGGTCTTGGTACCGTCC
*Blautia producta*	GAGAAAAAGGGGCAACAACA	TCAGCATCTTTTCCCCAATC
*Clostridium lactatifermentans*	GGTATCCCCCACGCTTATTT	CCTGCGGTCAATTCTTTGAT
*Clostridium glycyrrhizinilyticum*	CTGCAGATAACGCACAGGAA	GTACCGGCAGGCATATCTGT
*Oscillibacter valericigenes*	TGTGAGTCCCAGCTCTACGA	GCAGGGCCTCTCTCCATTTG
*Megamonas hypermegale*	ATTCGCCTTCACGACAATTC	TTACGATATCGGCGAGAACC
*Bacteroides caecicola*	TATACCATGCACGATGAACC	AAATACCTTCTTCCCTCACG

## Data Availability

The generated raw sequence dataset is available and was deposited in the NCBI Short Read Archive database under BioProject accession number PRJNA718522. All remaining data generated or analysed during this study are included in this published article and its supplementary information files.

## Supplementary Materials

The following are available online at https://www.mdpi.com/article/10.3390/microorganisms9071480/s1, Table S1. OTU table of caecal and eggshell microbiota. Table S2. OTU table of feed microbiota and chicken caecum microbiota fed conventional or sterile feed. Table S3. Real-time PCR quantification of 7 strains from phylum Firmicutes used for spraying of eggs on day 1 and/or day 20 of embryonic development.
